# Association between 30-s Chair Stand-Up Test and Anthropometric Values, Vibration Perception Threshold, FHSQ, and 15-D in Patients with Type 2 Diabetes Mellitus

**DOI:** 10.3390/biology10030246

**Published:** 2021-03-22

**Authors:** Francisco Javier Domínguez-Muñoz, Jorge Carlos-Vivas, Santos Villafaina, Miguel A. García-Gordillo, Miguel Ángel Hernández-Mocholi, Daniel Collado-Mateo, Narcis Gusi, José C. Adsuar

**Affiliations:** 1 Physical Activity and Quality of Life Research Group (AFYCAV), Faculty of Sport Science, University of Extremadura, 10003 Cáceres, Spain; fjdominguez@unex.es (F.J.D.-M.); svillafaina@unex.es (S.V.); mhmocholi@unex.es (M.Á.H.-M.); ngusi@unex.es (N.G.); 2 Health Economy Motricity and Education (HEME), Faculty of Sport Science, University of Extremadura, 10003 Cáceres, Spain; jorge.carlosvivas@gmail.com (J.C.-V.); jadssal@unex.es (J.C.A.); 3 Facultad de Administración y Negocios, Universidad Autónoma de Chile, Sede Talca 3467987, Chile; 4 Centre for Sport Studies, Rey Juan Carlos University, Fuenlabrada, 28943 Madrid, Spain; danicolladom@gmail.com

**Keywords:** diabetes mellitus, sit-to-stand test, body fat-mass percentage, foot health, health-related quality of life, sensitivity threshold

## Abstract

**Simple Summary:**

Type 2 diabetes mellitus is a chronic global disease with a series of complications that lead to problems in the somatosensory system, the cardiovascular system, health-related quality of life, foot health, and even balance. These balance problems arise from deficits in lower limb strength, among other causes. The 30-s chair stand-up test is a test that evaluates leg strength and is an easy, quick, inexpensive, and predictive test of different parameters. How this test relates to health-related quality of life, vibration sensitivity threshold, and foot health has not been studied. This study tests the degree of the relationships of the 30-s chair stand-up test with the 15-dimensional (15-D) questionnaire, Foot Health Status Questionnaire (FHSQ), vibration sensitivity threshold, and body composition questionnaires.

**Abstract:**

Background: Type 2 diabetes mellitus (T2DM) is a chronic, worldwide disease affecting more than 400 million people. This pathology involves several associated problems, such as diabetic neuropathy complications, obesity, and foot problems, both in terms of health and sensitivity. Objective: The objective of this study was to explore the relationships of the 30-s chair stand-up test with the Foot Health Status Questionnaire (FHSQ), the vibration perception threshold (VPT), and the 15-dimensional (15-D) questionnaire in T2DM people. Methodology: Ninety participants with T2DM were assessed in terms of fat mass percentage, VPT, foot health, health-related quality of life (HRQoL), and the 30-s chair stand-up test. Results: The 30-s chair stand-up test was found to exhibit a moderate relationship with “physical activity” (rho = 0.441; *p* ≤ 0.001) and “vigor” (rho = 0.443; *p* ≤ 0.001) from FHSQ. The 30-s chair stand-up test was also found to be weakly associated with foot pain (rho = 0.358; *p* = 0.001), 15-D total score (rho = 0.376; *p* ≤ 0.001), “sleeping” (rho = 0.371; *p* < 0.001), and “depression” (rho = 0.352; *p* = 0.001). Conclusions: The 30-s chair stand-up test is associated with “physical activity”, “vigor”, and “foot pain” from the FHSQ and the 15-D questionnaire total score and its dimensions “sleeping” and “depression” in type 2 diabetes mellitus patients. Therefore, following the results obtained, qualified clinicians can use the 30-s chair stand-up test as a good tool for monitoring and managing type 2 diabetes.

## 1. Introduction

Type 2 diabetes mellitus (T2DM) is a metabolic disease characterized by fasting and postprandial hyperglycemia and is due to a progressive deficit of insulin secretion that is initiated after a process of insulin resistance [1]; it affects 415 million people worldwide [2]. Moreover, the T2DM prevalence is rising in both men and women. The International Diabetes Federation states that 46.2% of persons who are eligible for a diagnosis of T2DM are still undiagnosed. Likewise, the World Health Organization (WHO) estimates that approximately 642 million people in the world will suffer from DM by 2040 [2]. Moreover, the aging population could have a significant impact on DM prevalence [3].

People with DM usually suffer from several problems, such as neuropathy [4]. These complications have an impact on health-related quality of life (HRQoL). In this regard, there are several instruments to measure HRQOL, among which the 15-dimensional (15-D) questionnaire is one of the most important. This questionnaire has been previously administered in patients with T2DM [5,6]. Diabetic peripheral neuropathy leads to different types of foot problems, such as loss of sensation, which affect balance [7]. To address this, it is necessary to determine the problems related to the above aspects, such as foot health. For this purpose, there are different tools to measure foot health, including the Foot Health Status Questionnaire (FHSQ). This instrument has been used to assess the quality of the foot in people with foot-related diseases [8] or in patients with DM who have diabetes-related ulcers [9,10].

Due to the progression of the disease, sensitivity may decrease in the most distal parts of the body, and diabetic peripheral neuropathy can be diagnosed. This type of complication can affect nociceptive small-diameter fibers in the skin [11], even affecting the neuromotor fibers and producing muscle weakness. Thus, patients with DM have been shown to have less strength than people without DM in knee flexion-extension, specifically 17% in the flexion action and 14% in the extension action [12]. This could negatively affect the balance of these people, causing modifications in gait and posture patterns [13] and affecting the foot and ankle proprioception [14] and even foot sensitivity [15]. For these reasons, it is relevant to know the balance and strength performances in the DM population. There are different ways to evaluate sensitivity, and one such way is through the Vibratron II test that allows the assessment of the vibration perception threshold (VPT). Moreover, the loss of sensitivity has been related to problems in walking speed [16], fall risk [17], and mobility disability [18]. The lower-limb strength also plays a key role. There are numerous tests to check the strength of lower limbs, one of which is the 30-s chair stand-up test.

The 30-s chair stand-up test belongs to the senior-fitness test battery developed by Rikli and Jones [19]. It is an instrument that has demonstrated its criterion validity with lower-body strength [19]. It is a tool that has demonstrated safety; responsiveness; and intrasession, intersession, intraobserver, and interobserver reliability [19,20,21]. It is also a type of test that has no floor effect and has been used in a multitude of studies with older people [19,21]. This type of test is used together with other tests to evaluate the functional capacity of people, and depending on the test results, a person can be diagnosed as robust, pre-fragile, or fragile [22]. As for the population with type 2 DM, there is research where the test has been used [23,24], and the reliability and validity for this group have also been proven [25,26].

The 30-s chair stand-up test is a test with many advantages. There is a very fast learning curve for the evaluator; the materials needed to perform it are commonly used, easy to use, and inexpensive; and the test does not require professional personnel to carry it out [27]. The test is very fast since it does not require more than 1 min per evaluation.

Although previous studies have reported the relationship between anthropometrics, FHSQ, 15-D questionnaire, and VPT with other tests such as the timed up and go test (TUG) in T2DM patients [28], no studies have analyzed the relationship between these assessments and the 30-s chair stand-up test. Thus, this study aimed to explore the possible relationships between the 30-s chair stand-up test and anthropometrics, 15-D questionnaire, FHSQ, and VPT in patients with T2DM.

## 2. Materials and Methods

### 2.1. Study Design and Participants

To analyze the relationships between the 30-s chair stand-up test and the anthropometric characteristics, FHSQ, VPT, and HRQOL, a total of 90 people (34 females and 56 males) with T2DM participated in this cross-sectional study. Participants were recruited in a primary care center. The following inclusion criteria were considered: (a) people with T2DM diagnosed between 40 and 85 years old and (b) people giving their written consent. Furthermore, the exclusion criteria were as follows: (a) people diagnosed with type 1 DM, (b) people for whom high-intensity exercises are contraindicated or people suffering from any condition (like retinopathy, musculoskeletal injuries, significant equilibrium issues, or an increased risk of thrombus) that contraindicates such exercises, (c) people under psychotropic or neurotoxic treatment or exposed to neurotoxins (e.g., from industrial accidents or contact with toxic residues), (d) people receiving radiation therapy, (e) people under high risk of nondiabetic neuropathy (such as HIV, alcoholism, or uremia), (f) people highly exposed to whole-body vibration at work, and (g) people having participated in previous whole-body vibration studies. The data for the sample characterization can be found in Table 1 of the article by Domínguez-Muñoz et al. [28].

Prior to the start of participant recruitment, a sample size estimation was conducted. In this regard, a sample size of 88 people with T2DM achieved 87% power to detect a difference (with a significance level of 0.05) of −0.31 between the null hypothesis (correlation of 0.09) and the alternative hypothesis (correlation of 0.40 [29]) using a two-sided hypothesis test.

According to the ethical standards of the Declaration of Helsinki, the procedure was approved by the University’s Bioethics Committee (approval number: 44/2012). The study was conducted from February 2013 to June 2013. Before starting the study, all patients were informed of the procedures and signed an informed consent form.

### 2.2. Procedures and Assessments

#### 2.2.1. Demographic Information and Diabetes Status

Information regarding age and parameters related to the diagnosis of T2DM was collected from study participants. Furthermore, information about diabetes management was mainly extracted by the glycosylated hemoglobin (HbA1c) result, which was assessed through a blood extraction.

#### 2.2.2. The 30-s Chair Stand-Up Test

In this physical fitness test, participants began the test with their arms crossed at chest height and from a seated position. They had to get up and sit down as many times as possible during 30 s [19]. This test has shown excellent reliability in patients with T2DM (ICC > 0.90) [26].

#### 2.2.3. Anthropometric Measures

The Tanita Body Composition Analyzer BC-418 MA was utilized to study different parameters related to body composition through bioimpedance. Body mass index (BMI) was obtained through the formula weight divided by height squared.

#### 2.2.4. Foot Health Status Questionnaire (FHSQ)

To assess self-perception about foot health, the Foot Health Status Questionnaire (FHSQ) was utilized [8,30]. This is an eight-dimension questionnaire with each dimension scoring between 0 to 100 (where 0 is the worst and 100 the best possible foot health status). The dimensions are as follows: (1) foot pain, (2) foot function, (3) footwear, (4) general foot health, (5) general health, (6) physical activity, (7) social capacity, and (8) vigor. This instrument has been previously validated in different podiatric diseases [31,32] and has been used in healthy patients as a control group [33].

#### 2.2.5. Vibration Perception Threshold (VPT)

The Vibraton II instrument (Sensortek, Inc., Clifton, NJ, USA) was used to assess the VPT. This tool consists of a module that regulates the vibration and two units (one for each foot) where the vibration amplitude, vibration regulator, and four different switches are displayed. In this regard, two of the switches are used to turn the equipment on and adjust the amplitude. A third switch is used to send the vibration amplitude to the modules, whereas the last switch is used as a decoy so that the evaluated person always hears the same sound from the switch, independently of whether the vibration amplitude is changed from one to another module.

The dimensions of each module are 12.5 × 8.5 × 23.5 cm. A label for each module (module A and module B) was used to easily recognize them. To prevent vibrations from being transmitted through the floor, each module was placed on a carpet. Each cylinder (where participants had to place their big toe) vibrated at 120 Hz. Amplitude can be modified and is expressed as vibration units. Vibration units can be calculated by the following equation:

A = x^2^/2

where x is the vibration unit (vu) and A is the amplitude expressed in microns (μ). This instrument has shown excellent reliability in patients with T2DM [34].

#### 2.2.6. HRQoL

The 15-D questionnaire [35] was utilized to evaluate the health-related quality of life. This questionnaire is composed of 15 dimensions: mobility, vision, hearing, breathing, sleeping, eating, speech, excretion, usual activities, mental function, discomfort and symptoms, depression, distress, vitality, and sexual activity. Each dimension corresponds to a question with 5 possible answers. Each question is answered on a scale ranging from 1 to 5, with 1 being the best and 5 the worst. The total score of the questionnaire, which represents the health status, is reached by the sum of all dimensions that leads to a total score for the questionnaire, (1—full HRQoL; 0—death). Previous studies have used the 15-D questionnaire in patients with diabetes [36,37,38].

### 2.3. Statistical Analytics

SPSS 25 for Windows (SPSS Inc., Chicago, IL, USA) was used to conduct the statistical analyses. Data are presented as mean and standard deviation (SD) or median and interquartile range (IQR). Nonparametric tests were used following the results of the Kolmogorov–Smirnov test and after checking the distribution of the 30-s chair stand-up test. Thus, the Spearman correlation coefficient was used to establish correlations between the 30-s chair stand-up test and the other variables. To avoid type I error, the Bonferroni correction for multiple comparisons was applied with the significance level set at p being less than 0.001. Schober’s classification thresholds were followed [29] to interpret the correlation coefficient: 0.10 to 0.39, weak; 0.4 to 0.69, moderate; 0.70 to 0.89, strong; and ≥0.9, very strong. On the other hand, a linear regression analysis was performed to explain the health-related quality of life and the dimensions physical activity, vigor, and foot pain of the FHSQ questionnaire in relation to the 30-s chair stand-up test.

## 3. Results

Table 1 illustrates the Spearman’s correlation coefficients between the 30-s chair stand-up test and the anthropometric data. No significant correlations were found after applying the Bonferroni post hoc correction.

Table 2 reports the Spearman’s correlation coefficients in the comparison between the 30-s chair stand-up test, the VPT, and the FHSQ. Moderate significant correlations were reported between the 30-s chair stand-up test and physical activity (rho = 0.441; *p* ≤0.001) and between the 30-s chair stand-up test and vigor (rho = 0.443; *p* ≤ 0.001). A small association was also found between the 30-s chair stand-up test and foot pain (rho = 0.358; *p* = 0.001). Moreover, an association that was close to but did not reach statistical significance was found between the 30-s chair stand-up test and general foot health (rho = 0.322; *p* = 0.002).

Table 3 shows the Spearman’s correlation coefficient between the 30-s chair stand-up test and the health-related quality of life total score, as well as for each dimension. Weak direct correlations were found between the 30-s chair stand-up test and the 15-D total score (rho = 0.376; *p* ≤ 0.001), sleeping (rho = 0.371; *p* < 0.001), and depression (rho = 0.352; *p* = 0.001). Moreover, associations that were close to but did not reach statistical significance were found between the 30-s chair stand-up test and discomfort and symptoms (rho = 0.316; *p* = 0.002) and between the 30-s chair stand-up test and vitality (rho = 0.327; *p* = 0.002).

Table 4 shows the linear regression with the health-related quality of life and the dimensions physical activity, vigor, and foot pain of the FHSQ questionnaire in relation to the 30-s chair stand-up test.

## 4. Discussion

The present study explored the relationships between the 30-s chair stand-up test and anthropometrics, the 15-D questionnaire, the FHSQ, and the VPT in patients with T2DM. The 30-s chair stand-up test is commonly used as a lower-limb strength measurement [39]. Although lower-limb strength is associated with greater physical fitness and health status in several populations, to our knowledge, this is the first study that relates the 30-s chair stand-up test with anthropometrics, foot health status through the eight dimensions of the FHSQ questionnaire, the VPT, and the 15-D questionnaire in T2DM patients.

The results showed that the 30-s chair stand-up test is moderately correlated with “physical activity” and “vigor” from the FHSQ. These outcomes are supported by previous studies, which highlighted the existing direct association between lower-limb strength and the regular practice of physical activity [40,41]. A weak relationship was also found between the 30-s chair stand-up test and “foot pain”. However, the relationship between the 30-s chair stand-up test and the FHSQ dimensions had not been previously studied. In this regard, general foot health or the presence of foot pain plays an important role in the continuous practice of physical activity since people who suffer from poor foot health or higher foot pain could be limited in their usual physical activity practice. This could explain the relationship found in the present study, which revealed that higher foot pain is related to lower performance on the 30-s chair stand-up test. Thus, there is no doubt that foot health may affect the functionality, physical activity, practice, and the performance of daily activities and consequently affect people’s health-related quality of life and general health status [42].

A weak relationship was also found between the 30-s chair stand-up test and health-related quality of life, as the 15-D questionnaire total score showed. Although the association between the 30-s chair stand-up test and health-related quality of life has not been previously explored in T2DM patients, previous studies revealed an association between lower-limb strength and health-related quality of life in the elderly [43,44,45,46] and people with different pathologies [47,48]. Specifically, and independently of the population, studies showed that greater strength levels have been associated with better physical fitness and health-related quality of life, as well as greater performance in daily life activities. Moreover, previous studies investigating the relationship between strength and function have generally compared muscle strength with a global measure of function such as maximum walking speed, as opposed to the functional moments produced at the lower extremity joints during specific everyday activities [49,50,51]. Thus, it seems that muscle strength is key for everyday functioning since inadequate strength levels may limit people in carrying out daily activities safely and efficiently [44].

Additionally, our study revealed an indirect relationship between the 30-s chair stand-up test and sleep and depression. Similar results were reported by previous studies for the association with sleep [52] and the association with depression [43,53,54,55]. Regarding sleep, previous studies reported similar associations in older people [39,56,57]. Fex et al. [52] also reported that poor sleep quality is related to worse strength levels, general physical fitness, and physical function. This could be explained by the association of long sleep duration with weight gain, increased risk of metabolic complications, T2DM, and mortality [58,59,60]. Thus, it seems that sleep quality is more important than sleep quantity.

Regarding depression, previous research has revealed that greater physical fitness is related to lower depression levels [53,54,55], and regular practice of physical activity has been also related to lower depression levels [43,55] and a greater health-related quality of life [54]. This is in line with the results of our study.

Lastly, our results showed a possible weak association of the 30-s chair stand-up test with general foot health, discomfort and symptoms, and vitality. However, these findings could not be confirmed with significant outcomes. Thus, they represent an interesting direction for future research.

### 4.1. Clinical Implications

In the case where the relationships between the 30-s chair stand-up test and other evaluation parameters are confirmed, the doctor evaluating the T2DM patient could use the 30-s chair stand-up test as an initial screening test for possible foot health conditions or poor HRQoL. It is important to note that this would be a complementary tool, but it is necessary to refer patients to a specialist to confirm if they have these problems. Therefore, the 30-s chair stand-up test could be used as a preliminary examination method for other medical problems because it is low-cost, easy to apply, fast, and does not require special equipment.

### 4.2. Limitations

Some limitations of this study should be considered. Future studies should consider increasing the sample size of both men and women to obtain enough statistical power and be able to divide and analyze the data by gender. Moreover, it could be interesting to apply other alternatives to Bonferroni’s adjustment for more efficient control of the type I error since some authors consider it conservative [61].

## 5. Conclusions

In patients with type 2 diabetes mellitus, the 30-s chair stand-up test was moderately associated with the FHSQ foot health questionnaire dimensions “physical activity” and “vigor”, weakly associated with the dimension “foot pain”, and weakly associated with the total score of the 15-D health-related quality of life questionnaire and its dimensions “sleep” and “depression”. All these associations were statistically significant. The conclusions should be taken with caution given that there were more men than women in the sample and there was a wide age range.

## Figures and Tables

**Table 1:** Correlations between the 30-s chair stand-up test and the anthropometric data in type 2 diabetes mellitus people (*n* = 90).

	30-s Chair Stand-Up Test	
	Spearman’s Rho	*p* *
Age (years)	−0.314	0.003
Weight (kg)	0.136	0.202
Height (cm)	0.271	0.010
BMI (kg/m^2^)	−0.057	0.595
Fat Mass Percentage (%)	−0.296	0.005
Total Body Water (%)	0.297	0.004
Fat-Free Mass (%)	0.297	0.004
Basal Metabolic Rate (Kcal)	0.268	0.011

* *p* refers to the *p*-value of the Spearman’s correlation coefficient.

**Table 2:** Correlations between the 30-s chair stand-up test and the vibration perception threshold (VPT) and the Foot Health Status Questionnaire (FHSQ) dimensions in the type 2 diabetes mellitus population (*n* = 90).

	30-s Chair Stand-Up Test	
	Spearman’s Rho	*p* *
VPT (vu)	−0.199	0.060
Foot Health Status Questionnaire (FHSQ)		
Foot Pain	0.358	0.001
Foot Function	0.233	0.027
Footwear	0.081	0.446
General Foot Health	0.322	0.002
General Health	0.209	0.048
Physical Activity	0.441	<0.001
Social Capacity	0.267	0.011
Vigor	0.443	<0.001

VPT: vibration perception threshold; vu: vibration units.* *p* refers to the *p*-value of Spearman’s correlation coefficient.

**Table 3:** Correlations between the 30-s chair stand-up test and the 15-dimensional (15-D) quality of life questionnaire dimensions and the total score in the type 2 diabetes mellitus population (*n* = 90).

	30-s Chair Stand-Up Test	
	Spearman’s Rho	*p* *
15-D Total Score	0.376	<0.001
15-D Quality of Life Questionnaire (15-D)		
Mobility	0.272	0.009
Vision	0.272	0.009
Hearing	0.071	0.509
Breathing	0.097	0.365
Sleeping	0.371	<0.001
Eating	N/A	N/A
Speech	0.137	0.198
Elimination	0.286	0.006
Usual Activities	0.171	0.106
Mental Function	0.077	0.472
Discomfort and Symptoms	0.316	0.002
Depression	0.352	0.001
Distress	0.132	0.214
Vitality	0.327	0.002
Sexual Activity	−0.001	0.995

N/A: not applicable. * *p* refers to the *p*-value of Spearman’s correlation coefficient.

**Table 4:** Linear regression model explaining health-related quality of life and the dimensions physical activity, vigor, and foot pain of the FHSQ questionnaire (*n* = 90).

	Health-Related Quality of Life (R^2^ = 0.166)		
Variables	β	Standard Error	*p*
Constant	0.695	0.049	<0.001
30-s Chair Stand-Up Test	0.017	0.004	<0.001
	Physical Activity (R^2^ = 0.120)		
	β	Standard Error	*p*
Constant	41.852	11.388	<0.001
30-s Chair Stand-Up Test	3.367	0.971	0.001
	Vigor (R^2^ = 0.174)		
	Β	Standard Error	*p*
Constant	15.960	12.127	0.192
30-s Chair Stand-Up Test	4.446	1.034	<0.001
	Foot Pain (R^2^ = 0.147)		
	Β	Standard Error	*p*
Constant	48.272	10.577	<0.001
30-s Chair Stand-Up Test	3.518	0.902	<0.001

## Data Availability

Not Applicable.

## References

[1] American Diabetes Association (2021). Summary of Revisions: Standards of Medical Care in Diabetes-2021. Diabetes Care.

[2] Ogurtsova K., da Rocha Fernandes J.D., Huang Y., Linnenkamp U., Guariguata L., Cho N.H., Cavan D., Shaw J.E., Makaroff L.E. (2017). IDF Diabetes Atlas: Global estimates for the prevalence of diabetes for 2015 and 2040. Diabetes Res. Clin. Pract..

[3] Valdés S., Rojo-Martínez G., Soriguer F. (2007). Evolución de la prevalencia de la diabetes tipo 2 en población adulta Española. Med. Clin..

[4] Alberti K.G.M.M., Zimmet P.Z. (1998). Definition, diagnosis and classification of diabetes mellitus and its complications. Part 1: Diagnosis and classification of diabetes mellitus. Provisional report of a WHO consultation. Diabet. Med..

[5] Makrilakis K., Liatis S., Tsiakou A., Stathi C., Papachristoforou E., Perrea D., Katsilambros N., Kontodimopoulos N., Niakas D. (2018). Comparison of health-related quality of Life (HRQOL) among patients with pre-diabetes, diabetes and normal glucose tolerance, using the 15D-HRQOL questionnaire in Greece: The DEPLAN study. BMC Endocr. Disord..

[6] Karamanakos G., Costa-Pinel B., Gilis-Januszewska A., Velickiene D., Barrio-Torrell F., Cos-Claramunt X., Mestre-Miravet S., Piwoñska-Solska B., Hubalewska-Dydejczyk A., Tuomilehto J. (2019). The effectiveness of a community-based, type 2 diabetes prevention programme on healthrelated quality of life. The DE-PLAN study. PLoS ONE.

[7] Di Nardo W., Ghirlanda G., Cercone S., Pitocco D., Soponara C., Cosenza A., Paludetti G., Di Leo M.A.S., Galli I. (1999). The use of dynamic posturography to detect neurosensorial disorder in IDDM without clinical neuropathy. J. Diabetes Complicat..

[8] Bennett P.J., Patterson C., Wearing S., Baglioni T. (1998). Development and validation of a questionnaire designed to measure foot-health status. J. Am. Podiatr. Med. Assoc..

[9] Dunn J.E., Link C.L., Felson D.T., Crincoli M.G., Keysor J.J., McKinlay J.B. (2004). Prevalence of Food and Ankle Conditions in a Multiethnic Community Sample of Older Adults. Am. J. Epidemiol..

[10] Burns J., Wegener C., Begg L., Vicaretti M., Fletcher J. (2009). Randomized trial of custom orthoses and footwear on foot pain and plantar pressure in diabetic peripheral arterial disease. Diabet. Med..

[11] Thomas P.K. (1997). Classification, Differential diagnosis, and staging of diabetic peripheral neuropathy. Diabetes.

[12] Andersen H., Nielsen S., Mogensen C.E., Jakobsen J. (2004). Muscle strength in type 2 diabetes. Diabetes.

[13] Mustapa A., Justine M., Mohd Mustafah N., Jamil N., Manaf H. (2016). Postural Control and Gait Performance in the Diabetic Peripheral Neuropathy: A Systematic Review. Biomed Res. Int..

[14] Van Deursen R.W.M., Simoneau G.G. (1999). Foot and ankle sensory neuropathy, proprioception, and postural stability. J. Orthop. Sports Phys. Ther..

[15] Ducic I., Short K.W., Dellon A.L., Disa J.J. (2004). Relationship between loss of pedal sensibility, balance, and falls in patients with peripheral neuropathy. Ann. Plast. Surg..

[16] Allet L., Armand S., de Bie R.A., Golay A., Pataky Z., Aminian K., de Bruin E.D. (2009). Clinical factors associated with gait alterations in diabetic patients. Diabet. Med..

[17] MacGilchrist C., Paul L., Ellis B.M., Howe T.E., Kennon B., Godwin J. (2010). Lower-limb risk factors for falls in people with diabetes mellitus. Diabet. Med..

[18] Ward R.E., Boudreau R.M., Caserotti P., Harris T.B., Zivkovic S., Goodpaster B.H., Satterfield S., Kritchevsky S.B., Schwartz A.V., Vinik A.I. (2014). Sensory and motor peripheral nerve function and incident mobility disability. J. Am. Geriatr. Soc..

[19] Jones C.J., Rikli R.E., Beam W.C. (1999). A 30-s chair-stand test as a measure of lower body strength in community-residing older adults. Res. Q. Exerc. Sport.

[20] Gill S.D., De Morton N.A., Mc Burney H. (2012). An investigation of the validity of six measures of physical function in people awaiting joint replacement surgery of the hip or knee. Clin. Rehabil..

[21] Telenius E.W., Engedal K., Bergland A. (2015). Inter-rater reliability of the Berg Balance Scale, 30 s chair stand test and 6 m walking test, and construct validity of the Berg Balance Scale in nursing home residents with mild-to-moderate dementia. BMJ Open.

[22] Fried L.P., Tangen C.M., Walston J., Newman A.B., Hirsch C., Gottdiener J., Seeman T., Tracy R., Kop W.J., Burke G. (2001). Frailty in older adults: Evidence for a phenotype. J. Gerontol. Ser. A Biol. Sci. Med. Sci..

[23] Mendes R., Sousa N., Themudo-Barata J., Reis V. (2016). Impact of a community-based exercise programme on physical fitness in middle-aged and older patients with type 2 diabetes. Gac. Sanit..

[24] Senior H., Henwood T., Mitchell G. (2015). Investigating innovative means of prompting activity uptake in older adults with type 2 diabetes: A feasibility study of exergaming. J. Sports Med. Phys. Fitness..

[25] Alfonso-Rosa R.M., Del Pozo-Cruz B., Del Pozo-Cruz J., Sañudo B., Rogers M.E. (2014). Test-retest reliability and minimal detectable change scores for fitness assessment in older adults with type 2 diabetes. Rehabil. Nurs..

[26] Barrios-Fernández S., Pérez-Gómez J., Galán-Arroyo M.D.C., Señorán-Rivera J., Martín-Carmona R., Mendoza-Muñoz M., García-Gordillo M.Á., Domínguez-Muñoz F.J., Adsuar J.C. (2020). Reliability of 30-s Chair Stand Test with and without Cognitive Task in People with Type-2 Diabetes Mellitus. Int. J. Environ. Res. Public Health.

[27] Cobo A., Villalba-Mora E., Pérez-Rodríguez R., Ferre X., Escalante W., Moral C., Rodriguez-Mañas L. (2020). Automatic and Real-Time Computation of the 30-Seconds Chair-Stand Test without Professional Supervision for Community-Dwelling Older Adults. Sensors.

[28] Domínguez-Muñoz F.J., Adsuar J.C., Carlos-Vivas J., Villafaina S., Garcia-Gordillo M.A., Hernández-Mocholi M.Á., Collado-Mateo D., Gusi N. (2020). Association between TUG and Anthropometric Values, Vibration Perception Threshold, FHSQ and 15-D in Type 2 Diabetes Mellitus Patients. Int. J. Environ. Res. Public Health.

[29] Schober P., Schwarte L.A. (2018). Correlation coefficients: Appropriate use and interpretation. Anesth. Analg..

[30] Landorf K.B., Keenan A.M. (2002). An evaluation of two foot-specific, health-related quality-of-life measuring instruments. Foot Ankle Int..

[31] Bennett P.J., Patterson C., Dunne M.P. (2001). Health-related quality of life following podiatric surgery. J. Am. Podiatr. Med. Assoc..

[32] Landorf K.B., Keenan A.M., Herbert R.D. (2006). Effectiveness of foot orthoses to treat plantar fasciitis A randomized trial. Arch. Intern. Med..

[33] Tovaruela-Carrión N., López-López D., Losa-Iglesias M.E., Álvarez-Ruíz V., Melero-González G., Calvo-Lobo C., Becerro-De Bengoa-Vallejo R. (2018). Comparison of health-related quality of life between patients with different metatarsalgia types and matched healthy controls: A cross-sectional analysis. Sao Paulo Med. J..

[34] Domínguez-Muñoz F.J., Adsuar J.C., Villafaina S., García-Gordillo M.A., Hernández-Mocholí M.Á., Collado-Mateo D., Gusi N. (2020). Test-retest reliability of vibration perception threshold test in people with type 2 diabetes mellitus. Int. J. Environ. Res. Public Health.

[35] Sintonen H. (2001). The 15D Instrument of Health-Related Quality of Life: Properties and Applications. Annals of Medicine.

[36] Saarni S.I., Härkänen T., Sintonen H., Suvisaari J., Koskinen S., Aromaa A., Lönnqvist J. (2006). The impact of 29 chronic conditions on health-related quality of life: A general population survey in Finland using 15D and EQ-5D. Qual. Life Res..

[37] Kontodimopoulos N., Pappa E., Chadjiapostolou Z., Arvanitaki E., Papadopoulos A.A., Niakas D. (2012). Comparing the sensitivity of EQ-5D, SF-6D and 15D utilities to the specific effect of diabetic complications. Eur. J. Health Econ..

[38] Sagarra R., Costa B., Cabré J.J., Solà-Morales O., Barrio F., Pinel B.C., Ribas B.B., Abat C.C., Torrell F.B., Luján F.M. (2014). Coste-efectividad de la intervención sobre el estilo de vida para prevenir la diabetes tipo 2. Rev. Clin. Esp..

[39] Kutner N.G., Schechtman K.B., Ory M.G., Baker D.I. (1994). Older Adults’ Perceptions of their Health and Functioning in Relation to Sleep Disturbance, Falling, and Urinary Incontinence. J. Am. Geriatr. Soc..

[40] Leblanc A., Taylor B.A., Thompson P.D., Capizzi J.A., Clarkson P.M., Michael White C., Pescatello L.S. (2015). Relationships between physical activity and muscular strength among healthy adults across the lifespan. Springerplus.

[41] Siddiqui N., Nessa A., Hossain M. (2010). Regular physical exercise: Way to healthy life. Mymensingh Med. J..

[42] Aparicio García-Molina V., Carbonell Baeza A., Delgado Fernández M. (2010). Beneficios de la actividad física en personas mayores. Rev. Int. Med. Y Ciencias La Act. Física Y Del Deport..

[43] Budiman-Mak E., Conrad K.J., Roach K.E. (1991). The foot function index: A measure of foot pain and disability. J. Clin. Epidemiol..

[44] Samuel D., Rowe P., Hood V., Nicol A. (2012). The relationships between muscle strength, biomechanical functional moments and health-related quality of life in non-elite older adults. Age Ageing.

[45] Yang S., Li T., Yang H., Wang J., Liu M., Wang S., He Y., Jiang B. (2020). Association between muscle strength and health-related quality of life in a Chinese rural elderly population: A cross-sectional study. BMJ Open.

[46] Hörder H., Skoog I., Frändin K. (2013). Health-related quality of life in relation to walking habits and fitness: A population-based study of 75-year-olds. Qual. Life Res..

[47] Lima T.R.L., Guimarães F.S., Carvalho M.N., Sousa T.L.M., Menezes S.L.S., Lopes A.J. (2015). Lower limb muscle strength is associated with functional performance and quality of life in patients with systemic sclerosis. Brazilian. J. Phys. Ther..

[48] Sener U., Ucok K., Ulasli A.M., Genc A., Karabacak H., Coban N.F., Simsek H., Cevik H. (2016). Evaluation of health-related physical fitness parameters and association analysis with depression, anxiety, and quality of life in patients with fibromyalgia. Int. J. Rheum. Dis..

[49] Skelton D.A., Greig C.A., Davies J.M., Young A. (1994). Strength, power and related functional ability of healthy people aged 65–89 years. Age Ageing.

[50] Rantanen T., Guralnik J.M., Izmirlian G., Williamson J.D., Simonsick E.M., Ferrucci L., Fried L.P. (1998). Association of muscle strength with maximum walking speed in disabled older women. Am. J. Phys. Med. Rehabil..

[51] Syddall H.E., Martin H.J., Harwood R.H., Cooper C., Aihie Sayer A. (2009). The SF-36: A simple, effective measure of mobility-disability for epidemiological studies. J. Nutr. Health Aging.

[52] Fex A., Barbat-Artigas S., Dupontgand S., Filion M.-E., Karelis A.D., Aubertin-Leheudre M. (2012). Relationship between Long Sleep Duration and Functional Capacities in Postmenopausal Women. J. Clin. Sleep Med..

[53] Bautista J.C., Bautista J.E.C., Martínez E.R.G., Pinilla M.I., Daza K.D.R. (2011). Aptitud física en mujeres adultas mayores vinculadas a un programa de envejecimiento activo. Salud UIS.

[54] Sáez-Padilla J., Sierra-Robles Á., Tornero-Quiñones I., Espina-Díaz A., Carvajal-Duque P. (2020). Condición física relacionada con depresión y calidad de vida en personas mayores. Rev. Psicol. del Deport. Sport Psychol..

[55] Rodriguez-Hernandez M., Araya Ramirez F., Ureña Bonilla P., Wadsworth D.D., Solano Mora L.C. (2014). Aptitud Física y su Relación con Rasgos Depresivos en Personas Adultas Mayores que Realizan Actividad Física. MHSALUD Revista en Ciencias del Movimiento Humano y Salud.

[56] Whitney C.W., Enright P.L., Newman A.B., Bonekat W., Foley D., Quan S.F. (1998). Correlates of daytime sleepiness in 4578 elderly persons: The cardiovascular health study. Sleep.

[57] Foley D., Ancoli-Israel S., Britz P., Walsh J. (2004). Sleep disturbances and chronic disease in older adults: Results of the 2003 National Sleep Foundation Sleep in America Survey. J. Psychosom. Res..

[58] Chaput J.P., Després J.P., Bouchard C., Astrup A., Tremblay A. (2009). Sleep duration as a risk factor for the development of type 2 diabetes or impaired glucose tolerance: Analyses of the Quebec Family Study. Sleep Med..

[59] Patel S.R., Malhotra A., Gottlieb D.J., White D.P., Hu F.B. (2006). Correlates of long sleep duration. Sleep.

[60] Kripke D.F., Garfinkel L., Wingard D.L., Klauber M.R., Marler M.R. (2002). Mortality associated with sleep duration and insomnia. Arch. Gen. Psychiatry.

[61] Martínez Camblor P. (2012). Ajuste del valor-p por contrastes múltiples. Rev. Chil. Salud Pública.

